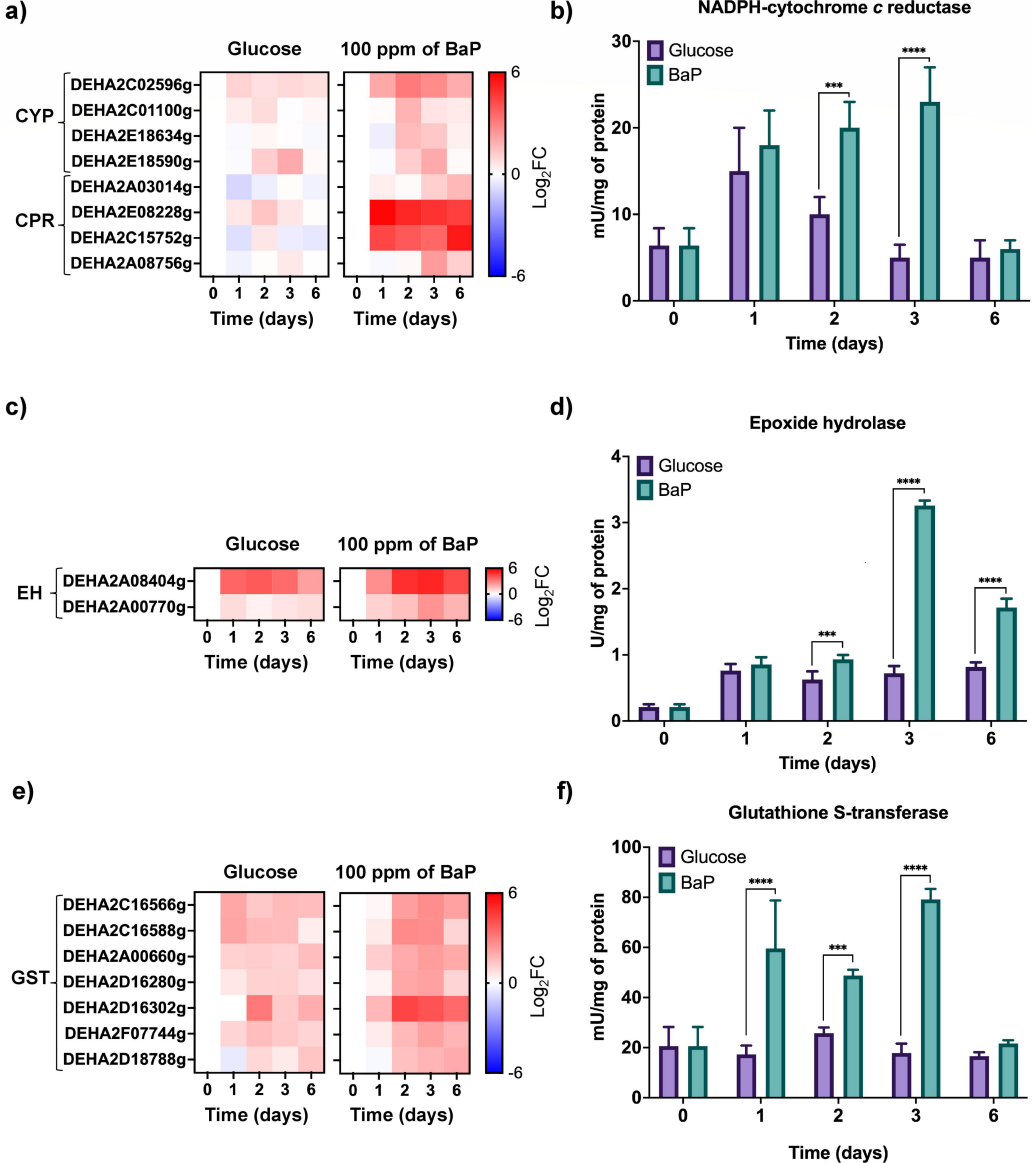# Articles of Significant Interest in This Issue

**DOI:** 10.1128/aem.00272-26

**Published:** 2026-02-18

## THE CO-EVOLUTION OF FUNGAL LACCASES AND PLANT LIGNIN

Liu et al. (e01971-25) describe the evolutionary history of laccase isozymes in white-rot fungi and demonstrate links to the ancestral emergence of their lignin substate in plants.


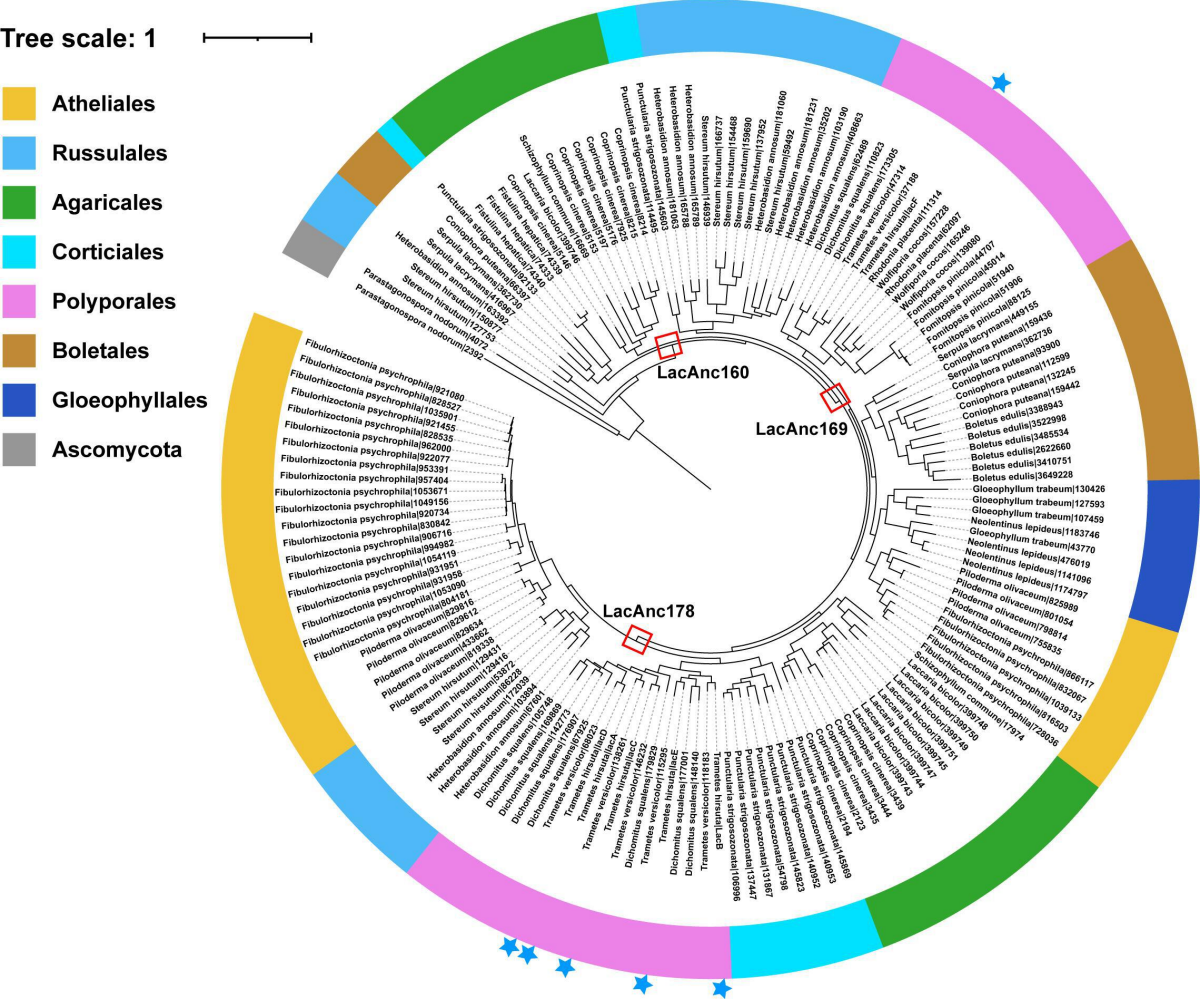


## A METAGENOMIC VIEW OF SEPTIC ARTHRITIS IN CATTLE 

This metagenomics survey of septic arthritis (SA) in cattle by Kos et al. (e01675-25) provides novel epidemiological insights into the etiological agents of SA and their antimicrobial resistance profile.


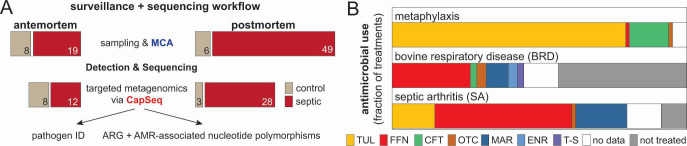


## *CLOSTRIDIOIDES DIFFICILE* STRESS RELIEF VALVE

Kalra et al. (e01988-25) describe conserved mechanisms for *Clostridioides difficile* survival under nitrosative stress in the colon that could guide therapeutic interventions against this recalcitrant pathogen.


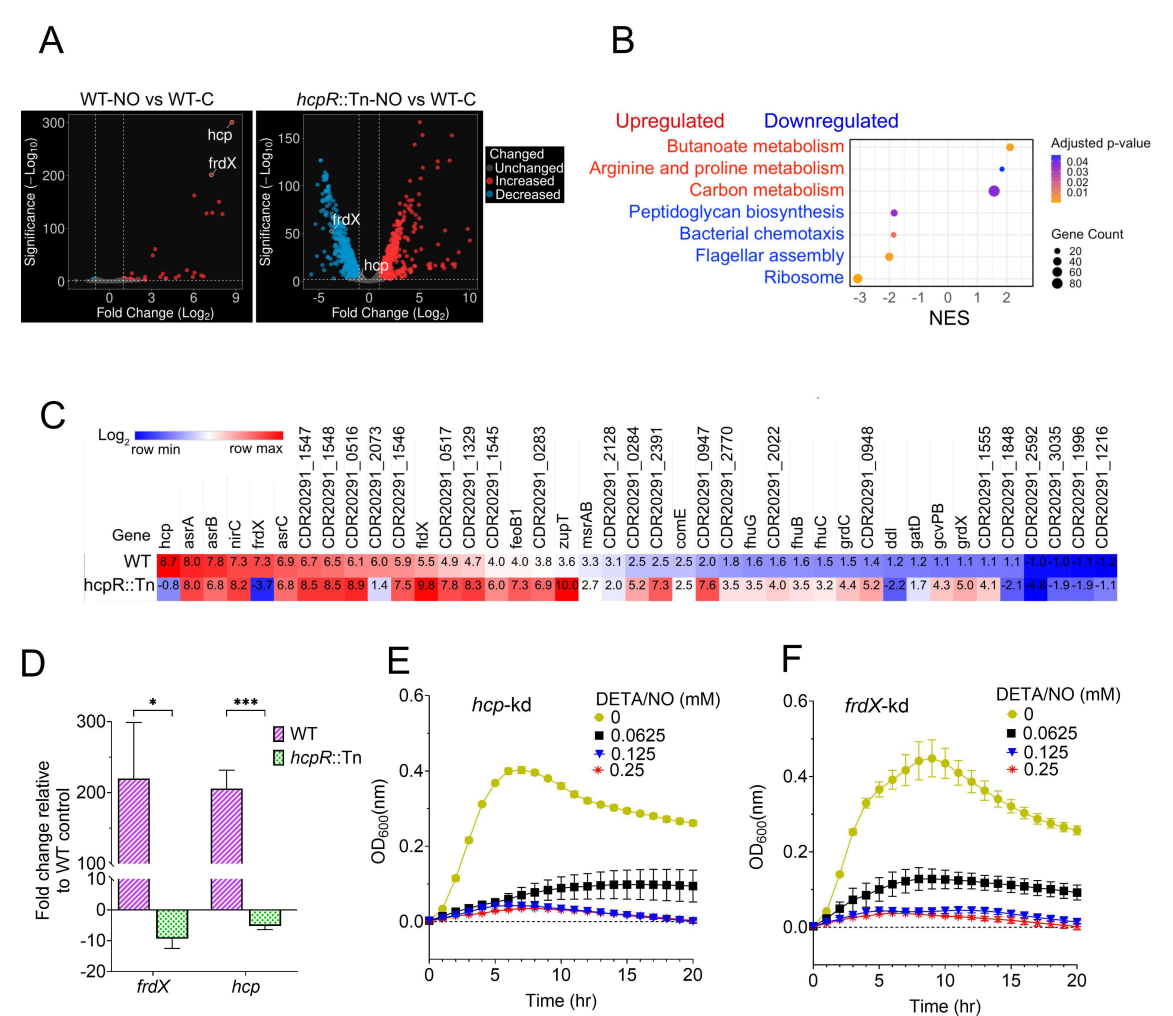


## A PHAGE COCKTAIL AGAINST *PSEUDOMONAS AERUGINOSA* 

Fujiki et al. (e02095-25) developed a phage cocktail against *Pseudomonas aeruginosa* that targets distinct classes of bacterial receptors to delay resistance evolution and enhance therapeutic robustness.


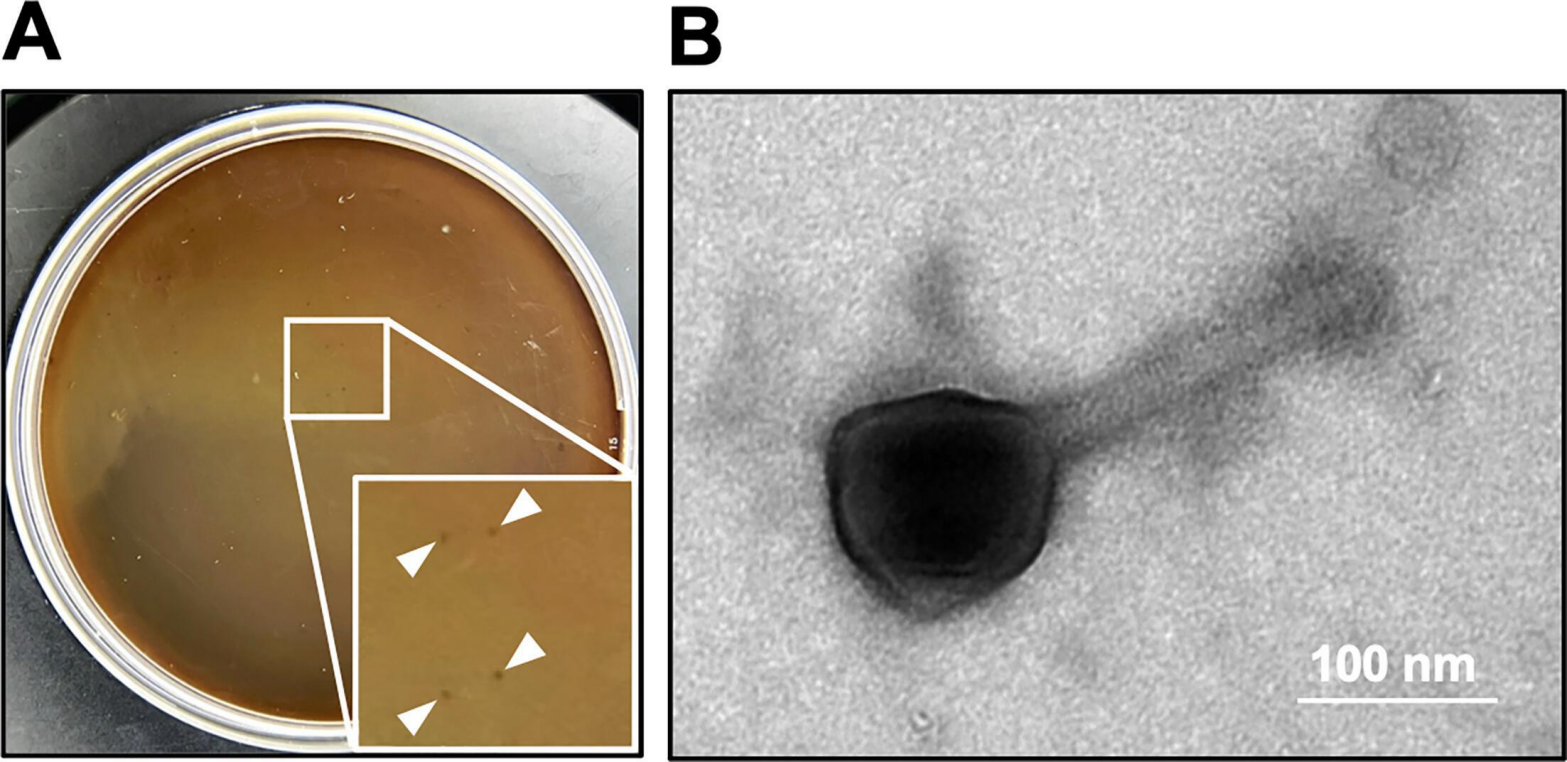


## MANUFACTURING PLATFORMS FOR FUNGAL CHEMICAL PRODUCTION 

Fungal terpene trichodiene can suppress the production of vomitoxin against the plant pathogen *Fusarium graminearum*. Hay et al. (e01695-25) describe a fermentation platform for scaled production of this important chemical.


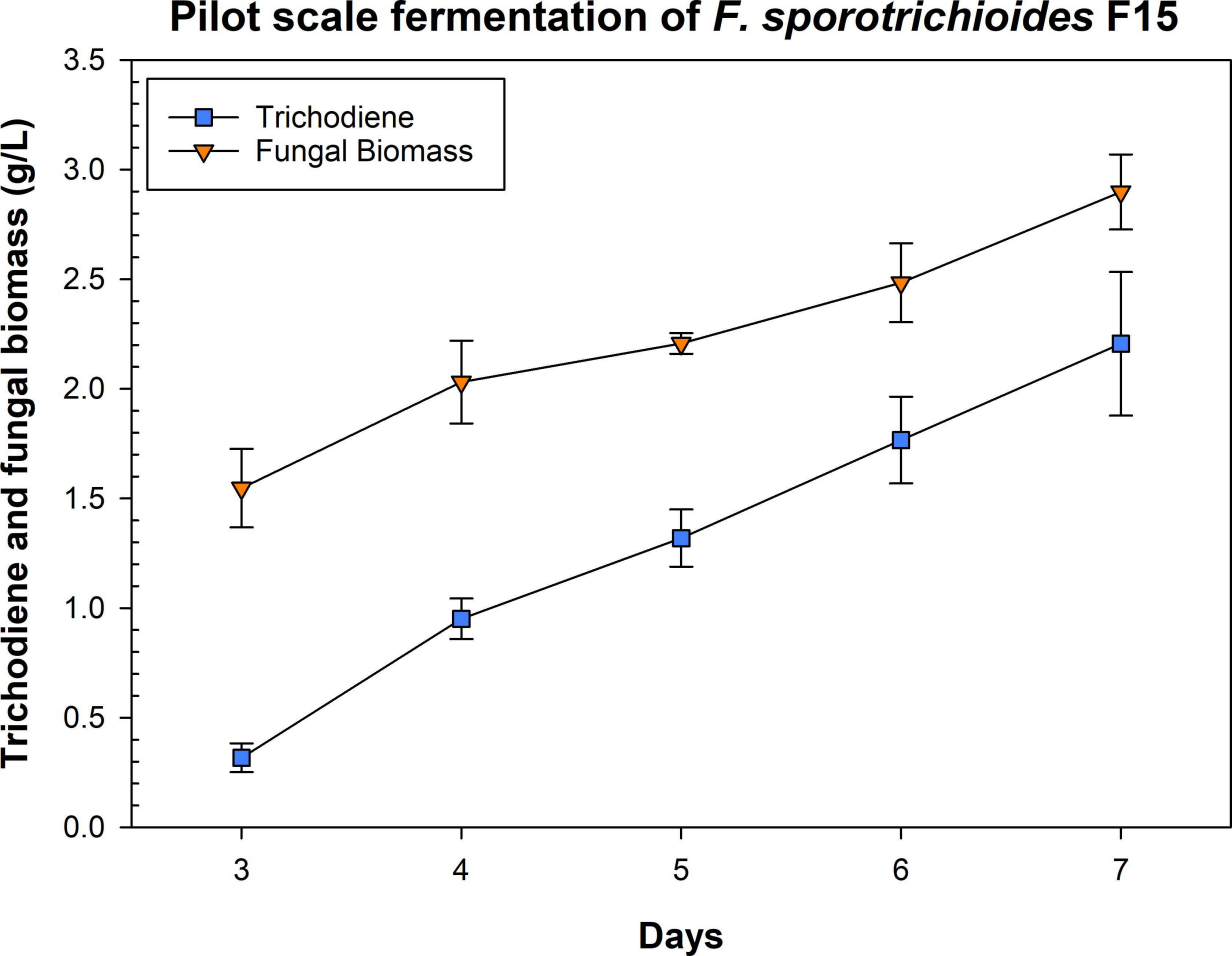


## A MARINE YEAST WITH A TASTE FOR AROMATIC HYDROCARBONS

Padilla-Garfias et al. (e02314-25) describe mechanisms for polycyclic aromatic hydrocarbons (PAHs) by a marine yeast, providing a foundational model to understand how eukaryotic microbes activate biotransformation pathways and antioxidant defenses under chemical stress.